# Spatiotemporal lake area changes influenced by climate change over 40 years in the Korean Peninsula

**DOI:** 10.1038/s41598-023-51084-2

**Published:** 2024-01-11

**Authors:** Myung Sik Cho, Jinwoo Park

**Affiliations:** 1 Center for Global Change and Earth Observations, Michigan State University, Lansing, MI USA; 2 Department of Geography and Geographic Information Science, University of North Dakota, Grand Forks, ND USA

**Keywords:** Environmental sciences, Hydrology

## Abstract

Water resources in lakes of the Korean Peninsula play a significant role in society and ecosystems in both South and North Korea. This study characterized spatiotemporal changes in the lake area during the dry season (March–May) in the Korean Peninsula over the last 40 years. The satellite images (Landsat 5–9) were used to derive annual areas of 975 lakes during the dry season from 1984 to 2023. Our analysis indicated that the MNDWI is the optimal remote sensing-based index for delineating lake areas in the Korean Peninsula, with an overall accuracy of 92.3%. Based on the selected index, the total lake areas of the dry seasons have increased from 1070.7 km^2^ in 1984 to 1659.3 km^2^ in 2023, mainly due to newly constructed dam reservoirs. While the detailed changes in lake area vary, we found divergent results based on their sizes. The large lakes (> 10 km^2^) showed their area increased by 0.0473 km^2^ (0.1%) every year and have more influences from climate change. On the contrary, the small lakes (≤ 10 km^2^) have area decreases by 0.0006–0.006 km^2^ (0.15–0.5%) every year and have less influence from climate change. This study shows that the spatiotemporal lake area changes are determined by either climate change or human activity.

## Introduction

Water resources in lakes are an essential component of the terrestrial hydrosphere. Lakes play a significant role in storing water during the wet season and releasing water during the dry season, especially for the region which has a large seasonal precipitation variability^1,2^. Many lakes have been constructed by damming the river and extracting groundwater to secure water resources and keep stable lake areas^3,4^. Meanwhile, drier climate conditions and water exploitation for water consumption, urbanization, and agriculture reduces water resources in lakes^5–7^. The efforts to secure water resources and threats to decrease water resources are compounded in lakes, so monitoring the lake individually can cause biased result^8–10^. Instead, monitoring a large number of lakes over the region in a long temporal scale helps understand the current status of regional water resources and establish a better policy^6,11–13^.

The remote sensing-based estimation of lake areas is one of the most optimal ways of monitoring lakes in a regional scale over time. Since the lake area is highly correlated with lake storage^7,11,14^ and the remote sensing data provides spectral information that can distinguish water pixels from others^15–18^, spatiotemporal analysis of lake areas is used to characterize water resources^6,12,13,19^. Various remote sensing methods have been developed to delineate water pixels, such as supervised classification^19^, unsupervised classification^20^, linear unmixing methods^21^, spectral transformation^22^, and spectral index method^15–18^.

Especially, the spectral index methods are widely used due to their ease of use and satisfactory results. This uses the distinct spectral characteristics of water pixels. For example, water absorbs most near-infrared (NIR) and shortwave infrared (SWIR) wavelengths, but it strongly reflects visible wavelengths (blue, green, and red)^15,16^. The normalized difference water index (NDWI) is the first spectral index method for water detection using NIR and green wavelengths^15^, and many indices subsequently have been developed based on the NDWI ^16–18^. The advantage of the spectral index methods is that they are usually designed to have a global threshold of 0 to separate water pixels (pixel value > 0) from non-water pixels (pixel values < 0). However, its performance significantly varies across regions and time, due to variations in scenes, atmospheric conditions, topographical characteristics, and water characteristics (e.g., turbidity)^23–25^. Adaptive thresholds were applied to overcome the limitations of global thresholding methods by examining the best thresholding values for specific regions^24,26^, but adaptive thresholds are still sensitive to the characteristics of surrounding pixels of water pixels^26,27^. Unfortunately, the results of the studies determining the most accurate remote sensing-based water index vary from region to region^23–28^. This implies that the water index should be regionally determined by considering the regional characteristics of landscape and water.

**Figure 1:**
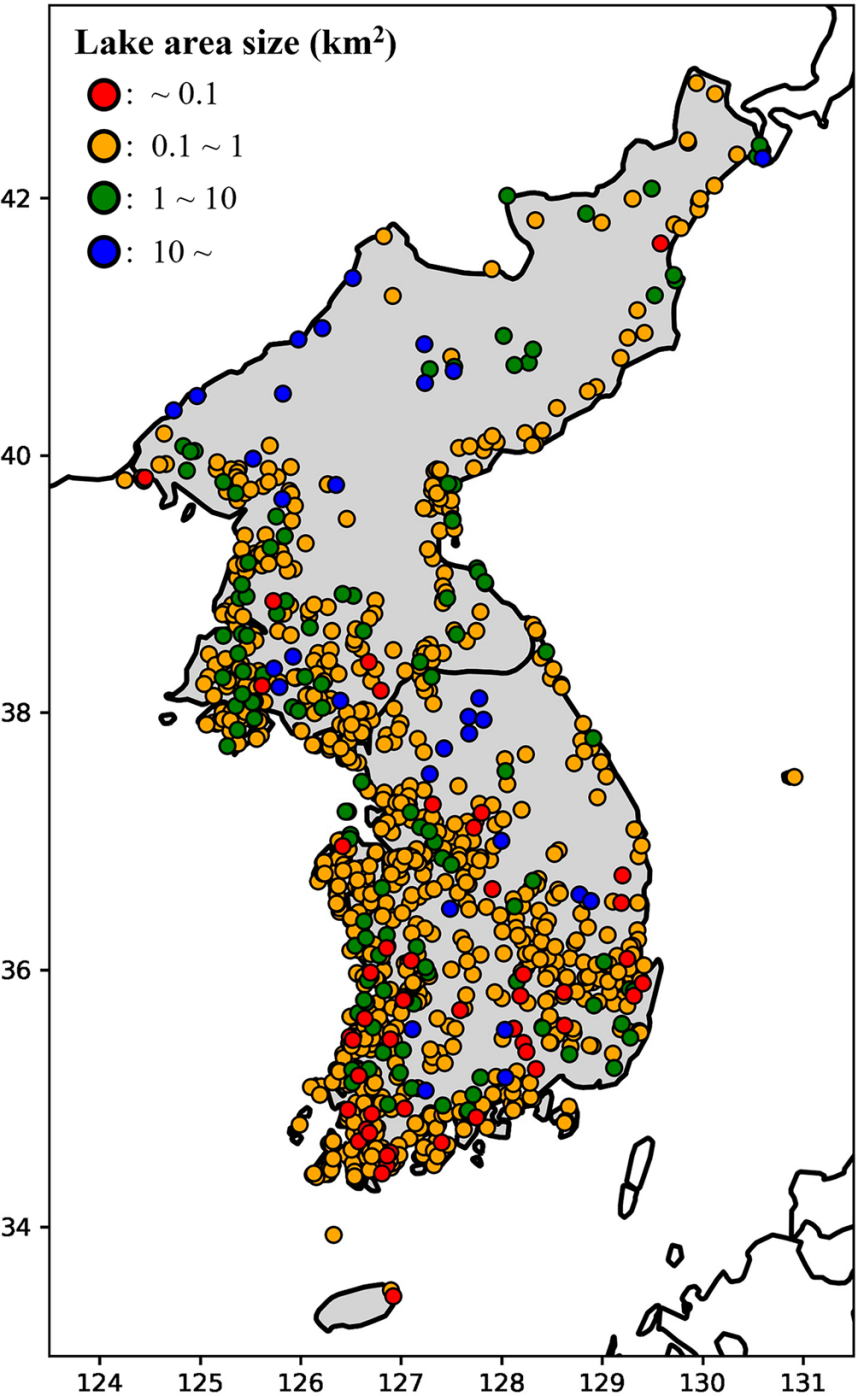
Locations of 975 Lakes in the Korean Peninsula groups by their sizes.

The remote sensing-based water index for the Korean Peninsula, where South Korea and North Korea are located, is necessary as lake areas have rarely been studied, although the location is under the pressure of climate change. The Korean Peninsula has over 975 lakes with a size over 0.1 km^2^ (Fig. 1; Messager et al. 2016). About 70% of precipitation is concentrated in summer (June through August) due to summer Monsoons and Typhoons, whereas its dry season is in winter and spring^29^. With the seasonal changes in precipitation, the lakes in the Korean Peninsula have large inter- and intra-annual variability. Because of the high seasonal variability in precipitation and large demands on water for the paddy rice fields (rice is the main staple), many small lakes were constructed across the Peninsula^19,30,31^. The tendency in the Peninsula that the dry season is drier, and the wet season is wetter has caused South Korea to increase the height of the agricultural reservoirs^30^. Additionally, the Korean Peninsula is under a distinct political system and the economic status between the two countries brings different lake management^30,32–35^. In this context, spatiotemporal lake area changes in the Korean Peninsula should be characterized for a better understanding of how lake areas have been changed under such a complex situation.

In this paper, we first determined the optimal remote sensing-based water index for the Korean Peninsula. We quantitatively compared the four popular indices with global threshold and adaptive threshold. Second, we characterized the lake area changes from 1984 to 2023 using 30 m spatial resolution Landsat 5–9 based on the selected index. The Korean Peninsula has suffered from severe hydrological drought during the dry season (March–May)^30^, so this study considered lake areas during the dry season. The annual areas of 975 lakes during the dry season over 40 years were individually extracted, and their spatiotemporal trends were quantified. Last, we determined the influence of climate change on lake areas using the Palmer drought severity index (PDSI), which can consider the complex influences of precipitation, temperature, and evapotranspiration on lakes^36^.

## Results

### The optimal remote sensing-based water index in the Korean Peninsula

The modified normalized difference water index (MNDWI) with global threshold, which refers to a threshold larger than 0 as a water pixel (see the details in **Methods**), outperformed other indices in delineating water pixels of lakes in the Korean Peninsula (Table 1). The MNDWI with a global threshold has an overall accuracy of 92.8%. For other indices using global threshold, the Automated Water Extraction Method (AWEI), Normalized Difference Water Index by McFeeter (${{\text{NDWI}}}_{{\text{M}}}$) and Normalized Difference Water Index by Rogers and Kearney (${{\text{NDWI}}}_{{\text{RK}}}$) have an overall accuracy of 91%, 89.5%, and 88.3% sequentially. For indices using adaptive threshold, the otsu method was used to define a scene-based threshold using spectral characteristics (see the details in **Methods**)^37^. The overall accuracy of ${{\text{NDWI}}}_{{\text{RK}}}$, MNDWI, ${{\text{NDWI}}}_{{\text{M}}}$ and AWEI are 74.1%, 72.5%, 70.6% and 68.3%.

**Table 1:** Accuracy results of delineating water pixels over four water indices with global and adaptive thresholds.

	Overall accuracy	Producer’s accuracy	User’s accuracy
Global threshold			
$NDW{I}_{M}$	89.5	62.1	99.1
$NDW{I}_{RK}$	88.3	60.2	98.1
*MNDWI*	92.8	78.9	97.6
*AWEI*	91.0	69.1	98.7
Adaptive threshold			
$NDW{I}_{M}$	70.6	96.3	61.6
$NDW{I}_{RK}$	74.1	95.7	66.6
* MNDWI*	72.5	97.6	63.7
* AWEI*	68.3	97.9	58.0

Indices using the global threshold were more accurate than those using the adaptive threshold in extracting water pixels of lakes in the Korean Peninsula (Table 1). The global thresholding methods have high user’s accuracy (97–99%), which indicates errors of commission and low producer’s accuracy (60–79%), which indicates errors of omission. On the other hand, the adaptive thresholding methods have low user’s accuracy (58–67%) and high producer’s accuracy (96–98%).

### Lake area changes in the Korean Peninsula

**Figure 2:**
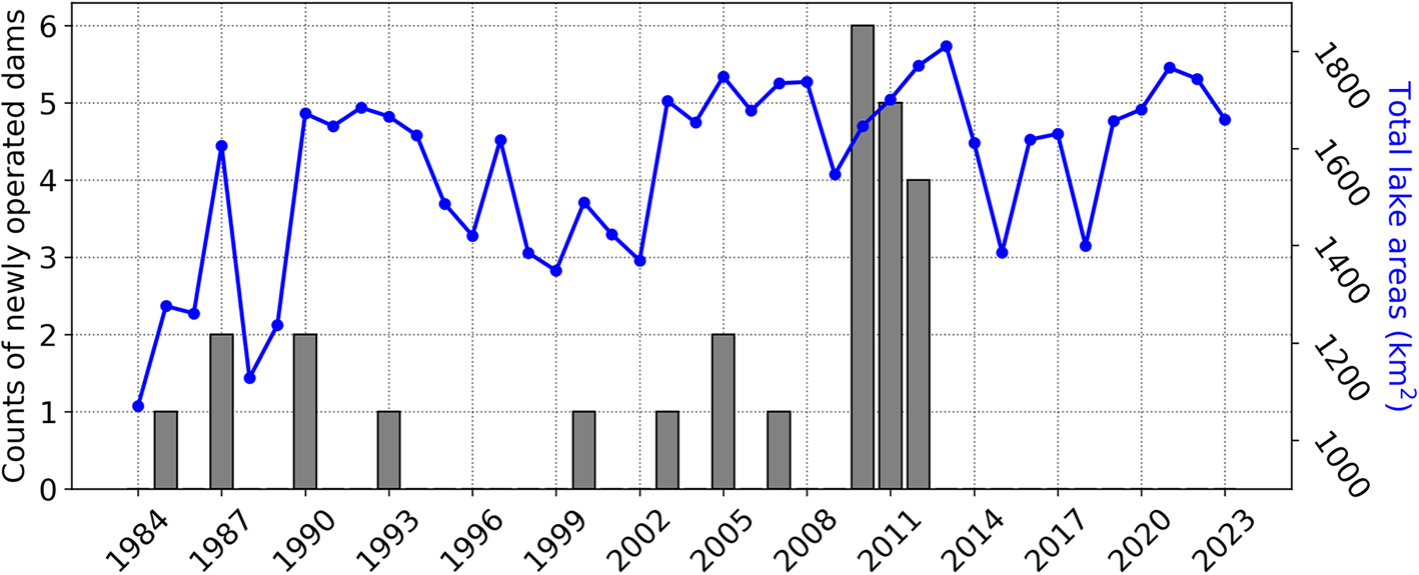
Total annual lake areas during dry season (blue color and right axis) and counts of newly operated dams which impound huge amount of water (grey color and left axis) from 1984 to 2023.

The total lake areas during dry season (March–May, see details in **Methods**) increased over the past 40 years in the Korean Peninsula (Fig. 2). In 1984, they were 1070.7 km^2^ and then were increased to 1659.3 km^2^ in 2023. The main factor in the increase was the construction of new lakes, especially dam reservoirs (grey bars in Fig. 2). Dams impound huge amounts of water, and 26 new dams have started their operations since 1984. In details, 1, 2, 2, 1, 1, 1, 2, 1, 6, 5 and 4 dams were newly operated in 1985, 1987, 1990, 1993, 2000, 2003, 2005, 2007, 2010, 2011 and 2012, respectively.

**Figure 3:**
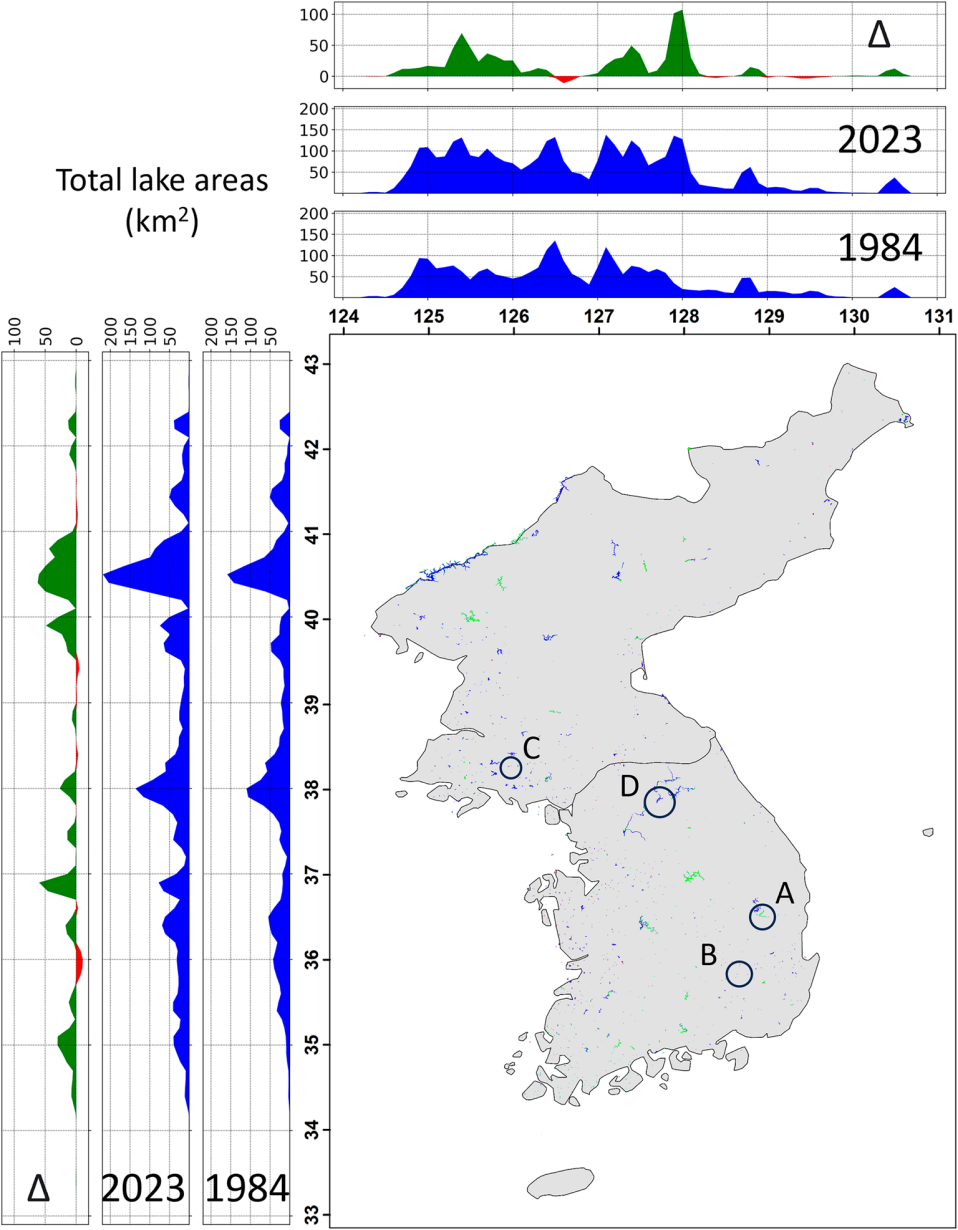
Lake locations and their area over latitudes and longitudes in 1984 and 2023 and their changes (Δ). The map demonstrates the water pixels of lakes observed in both 1984 and 2023 (blue), only in 1984 (red), and only in 2023 (green). Lake areas in 1984, 2023, and their differences (increased in green and decreased in red) are shown over every 0.1° in latitude and longitude in peripheral plots. Circles with **A, B, C**, and **D** indicate the locations of detailed maps in Fig. 4.

**Figure 4:**
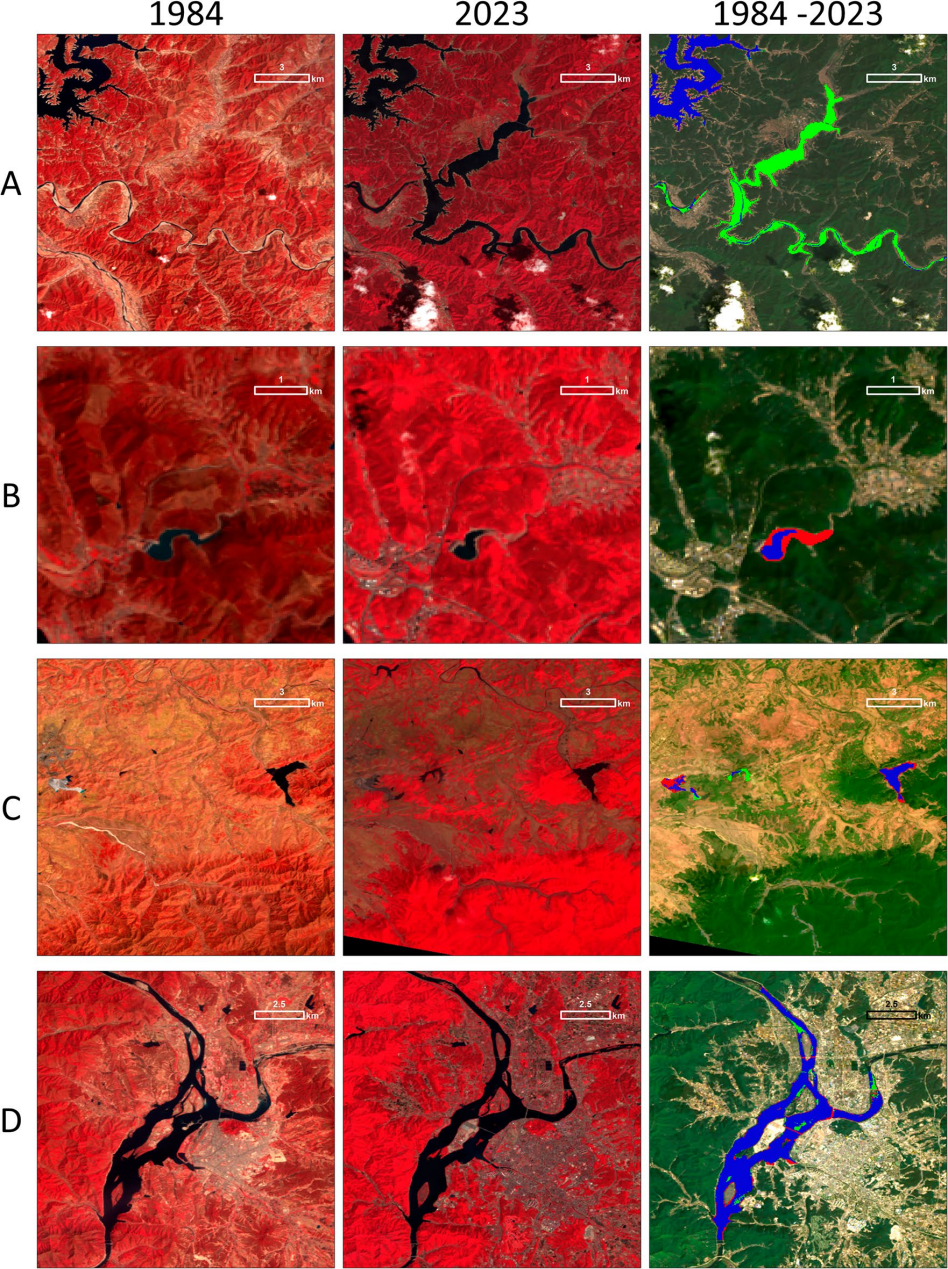
Detailed changes in lake area per subset in Fig. 3. Note: Rows represent the location of lakes. A: Imha Dam in Andong, South Korea; B: Lake Kongsan in Daegu, South Korea; C: Sariwon, North Korea; and D: Lake Uiam in Chuncheon, South Korea. Columns represent the time of each lake and their temporal differences. 1984 (Landsat 5; NIR-red-green color composite), 2023 (Landsat 9; NIR-red-green color composite), and changes in lake area from 1984 to 2023 (blue color: observed in both years, green color: gain, red color: loss).

The changes in the lake area varied from location to location. The newly built lakes largely contributed to increasing the lake areas (green plots and green areas in the map in Figs. 3 and 4A). Over the latitude, the area between 39.6° and 41° gained the largest amount of water by 472.4 km^2^ during the dry season over the course of 40 years. The areas around 38° and 36.9° and the ones between 34.2° and 35.6° increased lake areas by 36.3 km^2^, 105.8 km^2^ and 106.4 km^2^. Over the longitude, the area between 127° and 128.2° gained the largest amount of water between 1984 and 2023 by 451 km^2^. The areas around 128.8° and 130.5° and the one between 124.5° and 126.4° increased lake areas by 13.3 km^2^, 26.7 km^2^, and 421.3 km^2^ during the dry season. Imha dam (Fig. 4A) is an example of increased lake area (green color) due to a newly operated dam (commissioned in 1991), which impounded area along the river and made a lake.

On the contrary, human activity and climate change contributed to decreasing the lake areas (red plots and red areas in the map in Figs. 3 and 4B,C,D). Over the latitude, the area near 36° experienced the largest amount of water loss (25.9 km^2^). The areas around 38.4° and 39.3° and the ones between 41 and 41.7° lost lake areas by 4 km^2^, 10.7 km^2^, and 8 km^2^ during dry season, respectively. Over the longitude, the area around 126.6° showed the largest amount of water loss (20.5 km^2^). The area around 128.5° and the one between 129° and 130° also presented the lost of the lake areas by 27.4 km^2^ over the past 40 years. Figure 4B shows that the lake Kongsan decreased lake areas (red color) due to climate changes. As described in the later section in detail, this location shows the correlation coefficient between lake areas and the Palmer drought severity index (PDSI) over the lake is 0.59 (*p*-value < 0.05; see the details in **Methods**), which means that shrinking lake areas and drier climate during dry season are positively related. Sariwon, North Korea (Figure 4C) shows an example of decreasing lake areas due to expansion of agriculture. The northern part of the lake located to the left was converted to a rice paddy field (red color). The lake in the middle was built for storing water for irrigation (green color). The lake on the right lost water due to drier weather (red color) as its correlation coefficient with PDSI is 0.48 (*p*-value < 0.05). Figure 4D (lake Uiam) shows that urbanization could contribute to decrease lake areas. The sediment was deposited along the lake (red color), and was developed into urban infrastructure (road, building, etc.).

**Figure 5:**
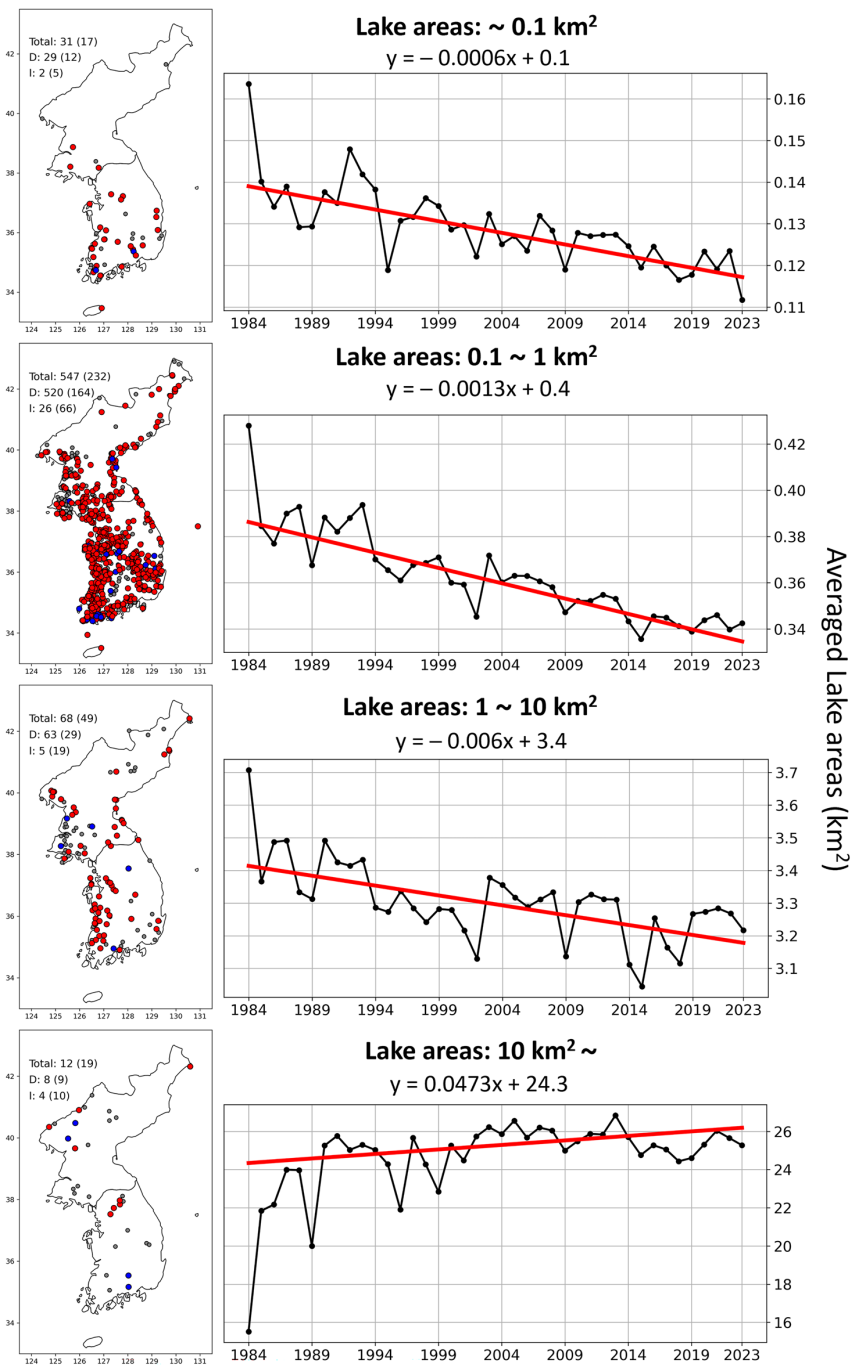
The trend of lakes area changing over the past 40 years per size. Note: The maps show the locations of the lakes along the statistical results (red: significant decrease (*p*-value < 0.05), blue: significant increase (*p*-value < 0.05), and grey: statistically insignificant). Texts in the maps indicate the number of lakes per size (without parenthesis), the number of statistically significant lakes (with parenthesis), and their trend (D: lakes with decreased area, I: lakes with increased area). The plots demonstrate the annually averaged lake areas with statistically significant results (both red and blue points on the maps) and their trends in black and red-colored lines, respectively.

Lake areas, during the dry season, changed differently by size during 1984–2023 (Fig. 5). The lakes with size smaller than 10 km^2^ experienced decreasing lake areas over the past 40 years, while the ones with size larger than 10 km^2^ gained lake areas (see the detailed criteria for grouping by size in **Methods**). Out of 48 lakes with a size smaller than 0.1 km^2^, 29 (60%) decreased lake areas and 2 (4%) increased lake areas statistically significantly (*p*-value of Mann–Kendall Test < 0.05; see the details in **Methods**). Each lake decreased lake area by 0.0006 km^2^ during the dry season every year. Among 779 lakes with a size larger than 0.1 km^2^ and smaller than 1 km^2^, 520 lakes (67%) decreased lake areas, and 26 lakes (3%) increased lake areas. Each lake decreased lake area by 0.0013 km^2^ during the dry season every year. Out of 117 lakes with a size larger than 1 km^2^ and smaller than 10 km^2^, 63 (54%) decreased lake areas and 5 (4%) increased lake areas. Lake areas of 0.006 km^2^ were shrunk every year. Among 31 lakes with a size larger than 10 km^2^, 8 lakes (26%) lost lake areas and 4 lakes (13%) gained lake areas. Lake areas of 0.0473 km^2^ were expanded every year.

### Influence of climate on lakes on the Korean Peninsula

**Figure 6:**
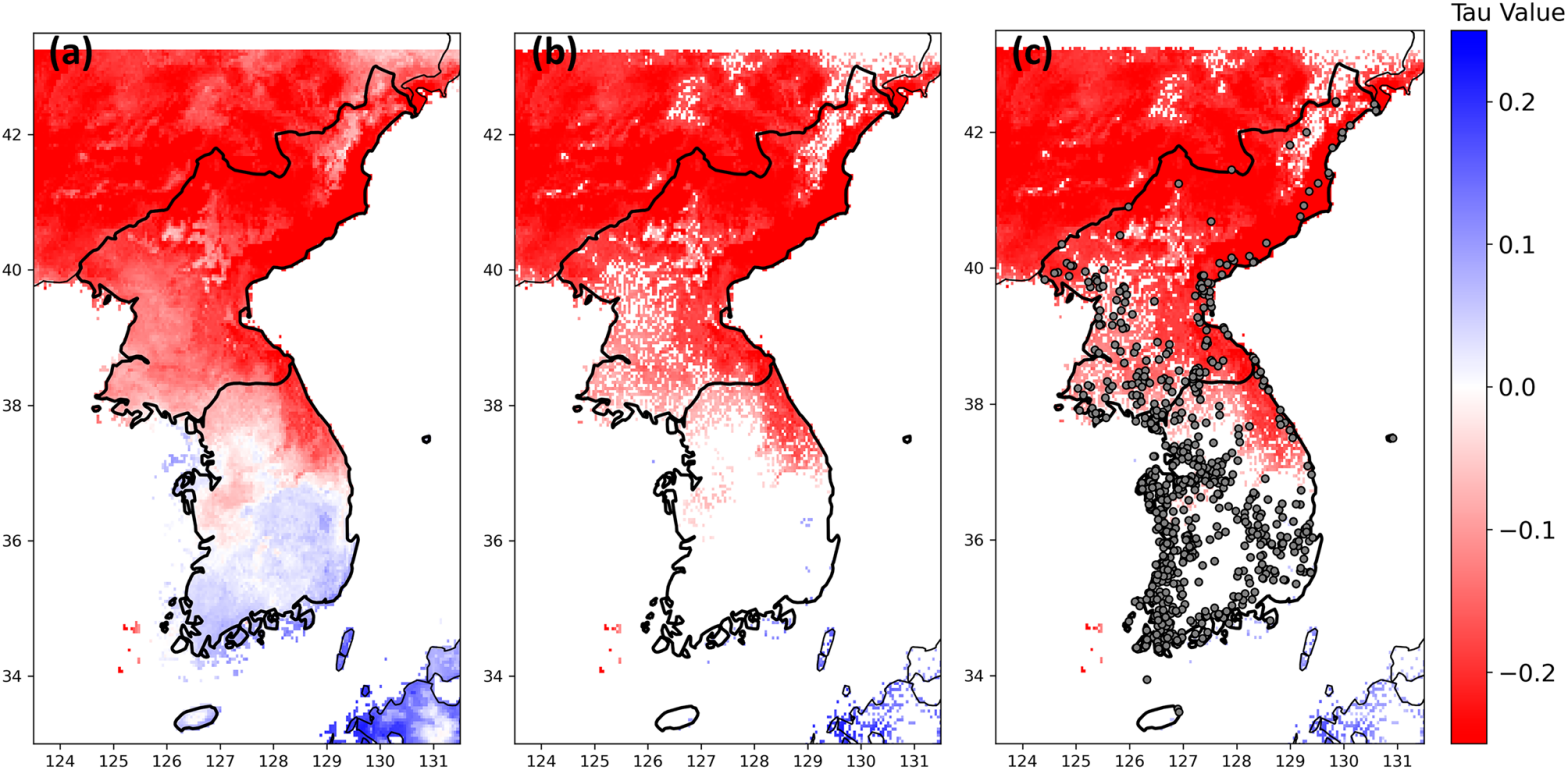
The magnitude maps for the trend of climate variability (Palmer Drought Severity Index; PDSI) from 1984 to 2023. The blue color represents a wetter trend, and the red color represents a drier trend. The tau value from Mann–Kendall Test **(a)** without consideration of *p*-value and **(b)** only significant *p*-value (< 0.05). **(c)** lakes with statistically significant changed areas (grey-colored points) were overlayed with (b).

Climate change significantly affected the Northern and Eastern part of the Korean Peninsula (Fig. 6; see the details in **Methods**). The climate had been drier (red color) during the dry season over the Northern and Eastern parts (Fig. 6b). Only small areas in the Southern part of the Korean Peninsula had been wetter (blue color) during the dry season. Half of the lakes were under drier climate conditions (Fig. 6c).

**Figure 7:**
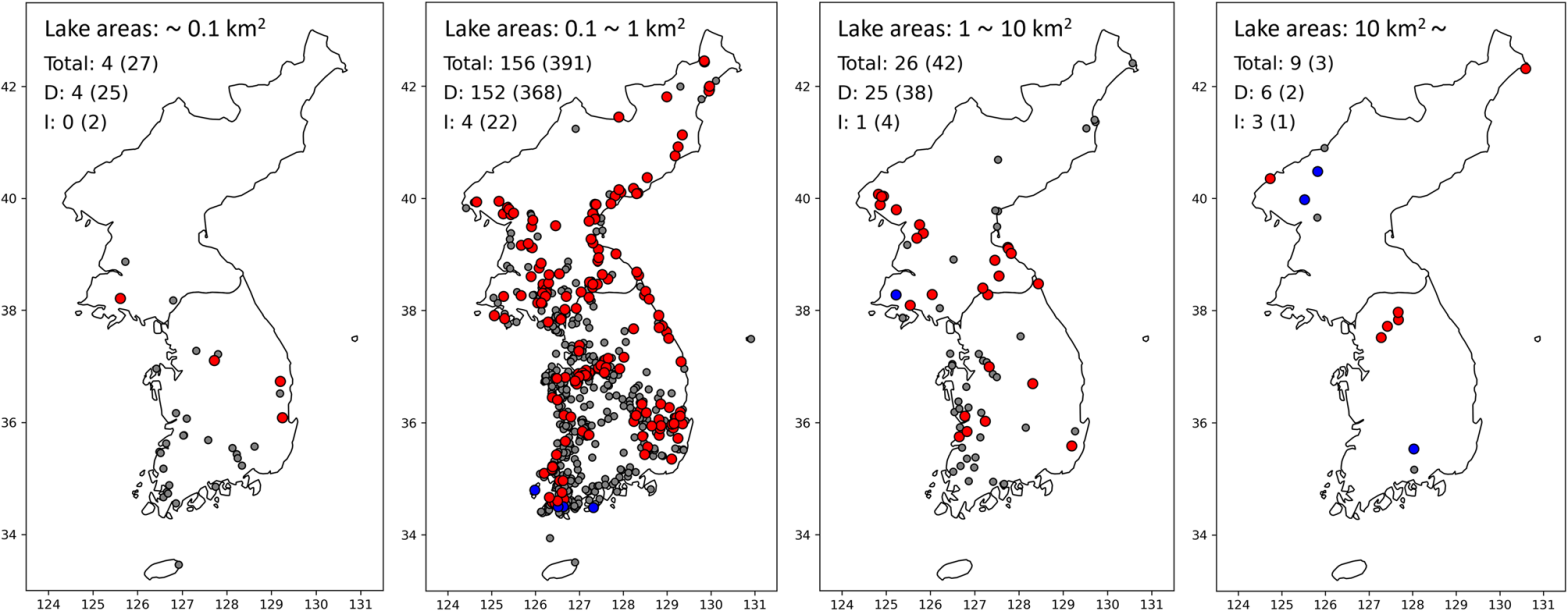
Locations of lake area changes which show positive correlations with climate variability (PDSI). Colors of the points indicate their trend in lake area changes along with the positive correlation with climate variability: red (decreased (*p*-value < 0.05)), blue (increased (*p*-value < 0.05) and grey (statistically insignificant). Texts in the maps indicate the number of lakes per size (without parenthesis), the number of statistically significant lakes (with parenthesis), and their trend (D: lakes with decreased area, I: lakes with increased area).

Smaller lakes in the Korean Peninsula are likely to have less influence from climate change, while changes in larger lakes could be attributed to (Fig. 7). Out of 31 lakes in the smallest category (with a size smaller than 0.1 km^2^), only 4 lakes (13%) have decreases in lake area related to climate change (PDSI; see the details in **Methods**). In addition, among 547 lakes with a size larger than 0.1 km^2^ and smaller than 1 km^2^, 152 lakes (28%) have decreases in lake area related to climate change, and 4 lakes (1%) have increases in lake area related to climate change. Out of 68 lakes with a size larger than 1 km^2^ and smaller than 10 km^2^, 25 lakes (37%) have decreases in lake area related to climate change, and 1 lake (1%) has increases in lake area related to climate change. Among 12 lakes larger than 10 km^2^, 6 lakes (50%) have decreased and 3 lakes (25%) have increased in their lake areas related to climate change.

## Discussion

This research presented that total lake areas during the dry season in the Korean Peninsula have increased since 1984. The smaller lakes (≤ 10 km^2^) showed a shrinking tendency, whereas it is less likely to be related to the drier climate conditions. The MNDWI was selected as an optimal remote sensing-based water index for extracting lake areas through the comparison with four well-known remote sensing-based water indexes and two threshold types. It was employed to delineate annual lake areas during the dry season (March–May) using Landsat 5–9. In short, the total lake areas during the dry season increased, mainly due to newly operated lakes, while climate change and human activity could have contributed to shrinking lake areas in our study area. Additionally, small lakes (≤ 10 km^2^) lose lake areas by 0.0006–0.006 km^2^ every year, while larger lakes (> 10 km^2^) gain lake areas by 0.0473 km^2^ every year. Notably, the increasing tendency of large lakes was mainly related to climate change, while the decreasing tendency of the small lakes was not.

### The optimal remote sensing-based water index in the Korean Peninsula

The complicated landscape surrounding lakes of South Korea and North Korea determines the MNDWI as the optimal water index for the Korean Peninsula (Table 1). Our result that the MNDWI outperformed is aligned with previous studies about its applications to China^25,28^, Australia^23^, and simulated scenes^26^. Most lakes in South Korea and North Korea are small reservoirs whose main purpose is agriculture^38^, and the scale of the agriculture is relatively small^39^. This makes lakes have complex landscapes with the composition of buildings, roads, and vegetation^40^. The MNDWI uses SWIR reflectance which can effectively distinguish the built-up areas and shadow from lower albedo features^16,17,23,25^. The MNDWI also utilizes green reflectance which can separate water from surrounding vegetation^16^.

Additionally, the complex landscape caused lower accuracy of the adaptive threshold. The otsu’s method, which was used for the adaptive threshold, finds the thresholds that can maximize the separability between the two classes (i.e., water and non-water)^37^. Thus, the otsu’s method can cause the misclassification for water bodies surrounded by complex features^23,27^. Contrarily, the global threshold can be robust to the complicated landscape, which has a variety of spectral range.

### Spatiotemporal characteristics of lake area changes in the Korean Peninsula

Our results indicated that the spatiotemporal characteristics in the lake area between the two countries (North and South Korea) were marginal during the dry season despite the systematic difference in their water-cycle patterns. In other words, the spatiotemporal changes were more influenced by the geographical locations than the water resource management (Fig. 3). To be specific, North Korea has pursued food self-sufficiency due to global restrictions from the United Nations for several decades^41,42^. Therefore, the country proceeded with deforestation, which has caused the loss of the ability to store water resources by converting forests to agricultural lands to effectively cultivate enough food^35,43^. For example, it cut trees and terracing with the hill slope over 16° over 2 million ha^43^. On the other hand, South Korea is one of the most successful countries of afforestation and systematically controls water resources over lakes^30,32^. Despite this difference in their water resource management, both countries frequently suffer from flooding and drought events. South Korea suffered from severe drought during the dry season due to climate change^30,31^, and North Korea has had frequent flooding and drought^33,34^.

Instead of the systematic differences between those two countries, our study detected more regional-scale spatiotemporal changes in lake areas, and those changes could be attributed to regional human activity and climate change (Fig. 3). For example, the construction of new lakes for generating electricity (Fig. 4A) and storing water for agriculture (Fig. 4B**)** largely increased water resources during the dry season. On the contrary, urbanization (Fig. 4D), expansion of agricultural land (Fig. 4C)**,** and drier climate conditions (Fig. 4B,C) significantly decreased water resources during the dry season. Additionally, water resources in small lakes (≤ 10 km^2^) are under pressure from human activity. The small lakes have been decreasing during the dry season over the past 40 years (Fig. 5), but their shrinkage is less likely to be related to climate changes (Fig. 7). In other words, human activity, such as urbanization and agriculture, can be a major factor in the decreasing lake areas in small lakes^13^. Due to urbanization and exploitation of water resources in South Korea^40^ and deforestation and expansion of agricultural lands in North Korea^34,41,43^, lake area changes of small lakes can be linked to human activity, rather than climate change.

### Current limitation and future direction

Despite our findings on the regional-scale spatiotemporal changes in the lake area and their distinctive relationship per size of the reservoir towards climate change, our study has a limitation on the climate change analysis. This is mainly attributed to the availability and quality of the input climate change data, given the absence of ground-truth climate change data (e.g., climate station), especially in North Korea. While the climate variability dataset (PDSI) is known to have one of the highest-resolution datasets among its competitors, its spatial resolution (4638 m) still has room to be improved. Our next study would increase the granularity of the climate change dataset with the climate station data in South Korea and apply it to North Korea. As our study revealed that there were marginal differences in the spatiotemporal changes in lake area changes between both countries, it would be interesting to see how climate change has affected the water resources in North Korea, especially since there are limited resources available in the country.

## Methods

### Data

The satellite data was used to extract the lake areas. Landsat 5–9 were used to detect lake areas from 1984 to 2023. Sentinel-2 was used to validate water detection from Landsat 5–9 for the period 2019–2022 due to the availability of satellite data. Images during the dry season (March–May) were selected for the lake every year to minimize the issues from the clouds and focus on lake areas during the dry season. The Landsat 5–9 Level 2 Collection 2 Surface Reflectance products provide 30 m spatial resolution and 16-day revisits from 1984. The Sentinel-2 Level 2 Surface Reflectance products provide 10 m spatial resolution for visible and NIR wavelength and 5-day revisits after the launch of Sentinel-2B in 2017.

All lakes (975 lakes) from the HydroLAKES over the Korean Peninsula were used for monitoring the lake changes (Fig. 1). The HydroLAKES dataset has 1,427,688 water bodies over 10 ha which were compiled from the eight global water products, including MOD44W and the Global Lakes and Wetlands Database (GLWD)^38^. Their global coverage and their ability to detect inland surface water made them widely used in water resource studies^44^. In addition, to increase the accuracy of our analysis, we manually check the dataset to remove misidentified locations (e.g., a location misidentified as a lake in HydroLAKES, while it is a river).

Palmer Drought Severity Index (PDSI) was considered for examining climate change because this can summarize the complex results of precipitation, temperature, and evapotranspiration, which affect surface water^36^. PDSI was derived from a gridded climate dataset, TerraClimate, which is based on climate observations and climate reanalysis dataset^45^. The size of the grid is 4638 m, so the grids over lakes were collected on a monthly scale during monthly scale. Despite its coarse resolution, the gridded climate dataset was used due to the insufficient number of climate stations over the lakes^46,47^, especially the locations in North Korea.

### Water pixel detection

The four popular spectral index methods with the global threshold and adaptive threshold were selected for finding the most optimal remote sensing-based water index for lakes in the Korean Peninsula. The ${MNDWI}_{M}$ is the first water index using green (562 nm) and NIR (865 nm) wavelengths developed by McFeeters **(1)**^15^. The ${MNDWI}_{RK}$ is the water index using red (655 nm) and SWIR (1650 nm) developed by Rogers and Kearney **(2)**^18^. The modified NDWI (MNDWI) is the water index using green (562 nm) and SWIR (1650 nm) **(3)**^16^. The automated water extraction index (AWEI) is the water index deriving from the empirical equations from the stable water areas globally using blue (482 nm), green (562 nm), NIR (865 nm), and SWIRs (1650 nm and 2215 nm) **(4–6)**^17^. The index is combined with ${AWEI}_{nsh}$ (for the non-shadow area) and ${AWEI}_{sh}$ (for the non-shadow area).

1 $${NDWI}_{M}= ({\rho }_{G}- {\rho }_{NIR})/({\rho }_{G}+ {\rho }_{NIR})$$

2 $${NDWI}_{RK}= ({\rho }_{R}- {\rho }_{SWIR1})/({\rho }_{R}+ {\rho }_{SWIR1}$$

3 $$MNDWI= {\rho }_{G}- {\rho }_{SWIR1})/({\rho }_{G}+ {\rho }_{SWIR1})$$

4 $$AWEI={AWEI}_{nsh} \bigcup {AWEI}_{sh}$$

5 $${AWEI}_{nsh}=4\times \left({\rho }_{G}- {\rho }_{SWIR1}\right)-\left(0.25\times {\rho }_{NIR}+2.75\times {\rho }_{SWIR2}\right)$$

6 $${AWEI}_{sh}={\rho }_{B}+2.5\times {\rho }_{G}-1.5\times \left({\rho }_{NIR}+ {\rho }_{SWIR1}\right)-0.25\times {\rho }_{SWIR2})$$

where ${\rho }_{B}$ is blue reflectance, ${\rho }_{G}$ is green reflectance, ${\rho }_{R}$ is red reflectance, ${\rho }_{NIR}$ is near-infrared reflectance, ${\rho }_{SWIR1}$ is SWIR (1650 nm), and ${\rho }_{SWIR2}$ is SWIR (2215 nm).

The index with the global threshold detects water pixels the values larger than 0. The index with the adaptive threshold detects water pixels using the otsu’s method. The otsu’s method is performing automatic image thresholding by maximizing inter-class variance and minimizing intra-class intensity variance^37^. By using Otsu’s method on the scenes calculated by indices, this paper separated water pixels and non-water pixels **(7–11)**.

7 $$P\left(i\right)= \frac{{n}_{i}}{N}$$

8 $${W}_{water}= \sum_{i>t}^{1}P\left(i\right) \space {\text{and}}\space {W}_{non-water}=1-{W}_{water}= \sum_{i=-1}^{t}P\left(i\right)$$

where $P\left(i\right)$ is the probability of the occurrence of *i*, ${n}_{i}$ is the number of pixels with value *i,* and *N* is the total number of pixels. ${W}_{water}$ is the cumulative probability for water pixels, ${W}_{non-water}$ is the cumulative probability for non-water pixels, and the sum of ${W}_{water}$ and ${W}_{non-water}$ should be 1. *t* is a threshold used in dividing water and non-water pixels, and its range is in between -1 and 1.

9 $$\mu = \sum_{i=-1}^{1}i\times P\left(i\right),\space {\mu }_{water}= \frac{\sum_{i>t}^{1}i\times P\left(i\right)}{{W}_{water}}, \space{\text{and}}\space {\mu }_{non-water}= \frac{\sum_{i=-1}^{t}i\times P\left(i\right)}{{W}_{non-water}}$$

10 $$g= {W}_{water}\times ({\mu }_{water}-{\mu )}^{2}+{W}_{non-water}\times ({\mu }_{non-water}-{\mu )}^{2}$$

11 $${t}_{otsu}=argmax\left(g\right) \space and \space \left\{\begin{array}{c}{Pixel}_{water} \space if \space i>{t}_{otsu}\\ {Pixel}_{non-water} \space if \space i\le {t}_{otsu}\end{array}\right.$$

where $\mu$ is the global mean values of the pixels in a scene, ${\mu }_{water}$ is the mean values of water-labeled pixels, and ${\mu }_{non-water}$ is the mean values of non-water-labeled pixels. *g* is the between-class variance, and the selected threshold (${t}_{otsu}$) would be the maximum value of *g.* This method can consider the local variants due to the environmental noise for sites and scenes and provide a better thresholding for each scene^26^.

### Comparison of remote sensing-based water indices

The four spectral index methods with the global threshold and adaptive threshold were compared to the accuracy of detecting lake areas in the Korean Peninsula. For the comparison, 1260 points were randomly generated over the 500 m buffered spatial boundary of lakes for 4 years (2019–2022). The reason for considering a 500 m buffered spatial boundary was to have enough number of water and non-water pixels. The Landsat 8, which is operational from 2019 to 2022, was used to detect water/non-water at 1260 points, and visual inspections were conducted to have reference information of water/non-water at 1260 points using Sentinel-2.

Three measures (overall accuracy, producer’s accuracy, and user’s accuracy) were used to compare the performances of indices and thresholding methods based on the visual inspection^48^. The overall accuracy shows how each index and thresholding method labels the pixel correctly **(12)**. This can represent the comprehensive performance of each water delineating method easily and efficiently. The producer’s accuracy shows the omission errors, which represent how each index and thresholding method does not mislabel the water pixels as non-water pixels **(13)**. This can indicate whether each method includes all water pixels. The user’s accuracy represents the commission errors, which show how each index and thresholding method does not mislabel the non-water pixels as water pixels **(14)**. This can tell us the performance of each method in excluding all non-water pixels.

12 $$Overall \; accuracy= \frac{{Pixel}_{WW}+{Pixel}_{NN}}{{Pixel}_{WW}+{Pixel}_{NW}+{Pixel}_{WN}+{Pixel}_{NN}}$$

13 $$Produce{r}'s\space  \,\,accuracy= \frac{{Pixel}_{WW}}{{Pixel}_{WW}+{Pixel}_{WN}}$$

14 $$User's\space \,\,accuracy= \frac{{Pixel}_{WW}}{{Pixel}_{WW}+{Pixel}_{NW}}$$

where ${Pixel}_{i,j}$ refers pixel values of *i* (pixel values of the Landsat-8) and *j* (visual inspection using Sentinel-2). *N* indicates non-water and *W* indicates water.

### Spatiotemporal analysis of lake areas from 1984 to 2023

The annual areas of 975 lakes during the dry season from 1984 to 2023 were derived from Landsat 5–9 using the MNDWI. The individual spatial boundary of lakes was delineated for each year, and then areas were calculated. Landsat imageries between March and May were selected if they intersected the 30 m buffered spatial boundary of lakes. The Landsat pixels were then stacked by the same year, and pixels with a QA value of cloud confidence over 33% were removed to reduce cloud effects. The stacked pixels, which are Landsat imageries from March to May of the same year, were aggregated into one pixel by taking the median value. The median pixel value provides the most robust pixel value of the dry season for a year. Then the MNDWI was applied to the median Landsat imagery to delineate the spatial boundary of lakes. Based on the boundary, the lake areas were calculated for a year for a lake from 1984 to 2023.

The Mann–Kendall trend test was conducted for individual lakes to examine the tendency of lake area changes over 40 years (Fig. 5)^49^. When the *p*-value is smaller than 0.05 and the tau value is positive, then the lake area is statistically significantly increasing. When the *p*-value is smaller than 0.05 and the tau value is negative, then the lake area change is statistically significantly decreasing. When the *p*-value is larger than 0.05, the lake area change is insignificant.

### Influences of climate changes on lake areas

The trend of PDSI was calculated by individual pixels to represent the tendency of climate change over the space (Fig. 6). The Mann–Kendall trend test was conducted, and the insignificantly changed pixels (*p*-value > 0.05) were removed (Fig. 6b).

The Spearman's rank correlation was conducted to determine the relationship between PDSI and lake areas. For this, the mean of the PDSI values during the dry season (March–May) over the lake location was extracted for every year. The Spearman's rank correlation was conducted for the collected annual PDSI values and the annual lake areas (Fig. 7).

## Data Availability

Upon a reasonable request, the data supporting this study's findings are available from the first author (Dr. Myung Sik Cho; chomyun2@msu.edu).
